# Synthesis, Characterization and Texture Observations of Calamitic Liquid Crystalline Compounds

**DOI:** 10.3390/ijms10114772

**Published:** 2009-11-04

**Authors:** Maher A. Qaddoura, Kevin D. Belfield

**Affiliations:** 1 Department of Chemistry; University of Central Florida, P.O. Box 162366, Orlando, FL 32816-2366, USA; E-Mail: mqaddour@mail.ucf.edu (M.A.Q.); 2 CREOL, The College of Optics and Photonics, University of Central Florida, P.O. Box 162366, Orlando, FL 32816-2366, USA

**Keywords:** liquid crystalline texture, nematic phase, smectic phase

## Abstract

Several divinylic mesogenic monomers were synthesized based on coupling the monomer 4-(4-pentenyloxy)benzoic acid with chlorohydroquinone, 2,5-dihydroxy- acetophenone, methylhydroquinone or 2-methoxyhydroquinone. This resulted in novel mesogens of phenylene esters with different lateral substituent groups. The effect of the lateral substituent group on the thermotropic phase behavior for these liquid crystalline compounds was investigated using DSC and optical polarized microscopy. All the mesogens proved to have a wide nematic liquid crystalline range. Only the phenylene ester, which has a methoxy lateral substituent, exhibited both nematic and smectic phases. Structural confirmation of all new derivatives was accomplished by ^1^H- and ^13^C-NMR spectroscopic analysis, along with CH elemental analysis.

## 1. Introduction

**Figure 1.**
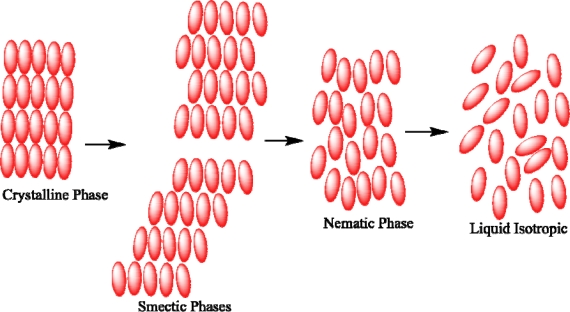
Phase transition between crystals, smectic, nematic, and isotropic liquid phases for a calamitic material as function of temperature.

Liquid crystalline behavior has been observed mainly in two types of molecules: linear or rod-like molecules that form calamitic phases and disc-like molecules that form discotic phases. The structure of liquid crystalline phases is characterized by the arrangement of the molecules, the conformation of the molecules, and the intermolecular interactions. The transition between the various liquid crystal mesophases from crystalline to smectic to nematic occurs at defined temperatures which can be detected using differential scanning calorimetry, while the nature and texture of the mesophase is normally investigated using polarized optical microscopy. The scenario involving phase transitions requires, as the first step, the breakdown of the molecular packing order under influence of an external heating source causing oscillation, or rapid rotation about the long axis of the molecule to give a ‘smectic-like’ crystal phase. Next, the long-range positional order is lost resulting in a smectic liquid crystal mesophase. Next, the local packing order is destroyed, but the orientational order still remains with the molecules reorganizing so that their long axes lie in the same direction producing the nematic phase. Finally, all order is lost forming an isotropic liquid[1]. The description of this melting process for rod-like molecules is shown schematically in Figure 1.

**Figure 2.**
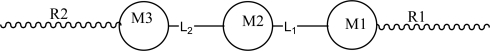
Model represents typical feature of calamitic liquid crystals, R1 & R2 are terminal groups, M1, M2 & M3 are ring systems, and L1 & L2 are linking groups.

Calamitic liquid crystals are characterized by their elongated, rod-like shape, and composed of three main structural elements: rigid ring systems, connective linkage groups, and flexible terminal groups. A general structure is given in Figure 2.

**Figure 3.**
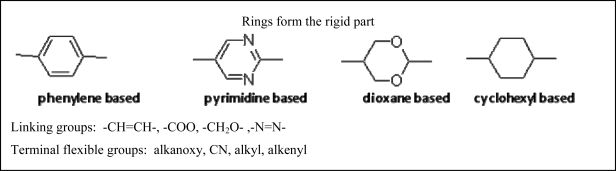
Examples of typical groups which represent the main constituents of calamitic liquid crystalline molecules.

Here, R1 and R2 are terminal groups, M1, M2, and M3 are ring systems and usually two to four of these units are present; they could be identical or different groups, and, finally, L represents linking groups. The most common of these, along with typical rings and linking groups, are listed in Figure 3 [2]. A wide range of lateral substituents (*e.g.*, F, Cl, CN, NO_2_, CH_3_, CF_3_) have been incorporated into many liquid crystal structures; a lateral substituent is one that is attached off the linear axis of the molecule, usually on the side of an aromatic core or even alicyclic moieties. The presence of the lateral substituents leads to disruption of molecular packing and, therefore, reduces liquid crystalline phase stability. This disruption of the molecular packing is particularly advantageous for the mesomorphic and physical properties required for specific applications. In general, the depression of the clearing point expressed as T_N-I_ by a lateral substituent is directly proportional to the substituent size, irrespective of its polarity [2].

Polarized light microscopy is commonly used to identify the texture of liquid crystalline mesophases. The textures are pictures that are observed microscopically under polarized light and characterized by defects of the phase structure that are generated by the distortion of the phase structure by the surrounding glass slides and coverslips (surface phenomena). Therefore, several different textures can be observed for a given structure depending on the particular conditions of sample preparation [3]. For example, an isotropic liquid will be dark under crossed polars, while birefringent liquid crystals exhibit interference colors.

A thin sample of the nematic phase, when viewed under polarizing microscopy, is often seen optically extinct, and appears dark. However, there may be small areas of a birefringent texture with dark (optically extinct) thread-like defects if the sample is thick. Displacement of the coverslip (shearing) induces homogenous (planar) alignment, producing a temporary birefringent flash. On heating the sample, homogeneous alignment is often induced close to the clearing point. It is at this point that the phase appears most colorful (schlieren texture) and a thin sample looks particularly sharp and is readily imaged [2,4].

When cooling an isotropic liquid, birefringent droplets appear against a dark background as the nematic phase is generated. In thick samples, these droplets coalesce to give a schlieren texture that may contain the thread-like defects. On cooling, the nematic phase appears from the isotropic liquid and so the texture that results is called a natural texture.

Calamitic liquid crystals based on p-alkoxy benzoic acid were investigated earlier for their liquid crystalline properties [5,6], and recently gained increased interest for the design of many liquid crystalline compounds. Demus *et al.* were among the first researchers to use calamitic liquid crystals for practical applications in the LCD industry [7–10]. However, the terminal end of the compounds were unfunctionalized linear aliphatic chains. On the other hand, alkenyloxy benzoate monomers are a functionalized version of alkoxy benzoate, a construct that also gained special attention in the design of functionalized liquid crystalline materials [11–15].

Reported herein are divinylic calamitic liquid crystals that are based on functionalized alkenyloxy benzoates coupled with dihyroquinone derivatives. These represent attractive functionalized compounds for use as precursors to design more advanced materials, *e.g.*, when crosslinked with siloxanes. This type of versatile crosslinking may result inunique liquid crystalline polymers and elastomers with potential applications in the field of artificial muscles [16], separation membranes, optical materials, bifocal contact lenses [17–19], holography [20], drug delivery [21], and electrooptics [22]. We have designed and synthesized a series of functionalized divinylic calamitic liquid crystals with different lateral substituents. These materials have been fully characterized, including their thermal behavior and texture observations.

## 2. Results and Discussion

### 2.1. Synthesis of 4-(4-Pentenyloxy)benzoic Acid

**Scheme 1.**
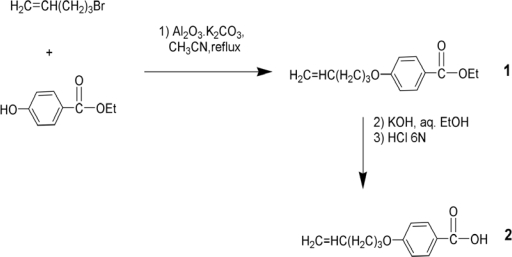
Synthesis of 4-(4-pentenyloxy)benzoic acid).

The synthesis of alkenyloxycarboxylic acid **2** is shown in Scheme 1; ethyl 4-hydroxybenzoate was etherified with 5-bromo-1-pentene in the presence of a catalyst system comprised of potassium carbonate supported on alumina. This system gave a high yield of product **1** with a minimum reaction time of 4 h. This is significant since many researchers have used different catalyst systems, such as K_2_CO_3_ in acetone or KOH in ethanol, producing lower yields and requiring longer reaction times (typically 12–48 h) [23–27]. ^1^H and ^13^C spectra were consistent with the proposed structure for **1**.

### 2.2. Synthesis and Thermotropic Behavior of Benzoate Mesogenic Compounds

**Scheme 2.**
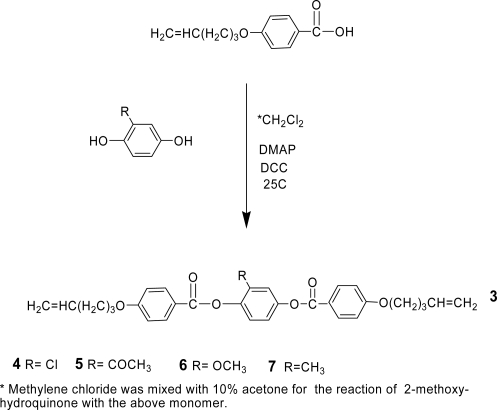
Synthesis of the calamitic liquid crystalline structures.

Four mesogenic structures, having the general structure **3** but with different lateral substituents, have been synthesized as shown in Scheme 2.

#### 2.2.1. Benzoic Acid, 4-(4-Pentenyloxy)-, 2-Chloro-1,4-phenylene Ester (**4**)

**Figure 4.**
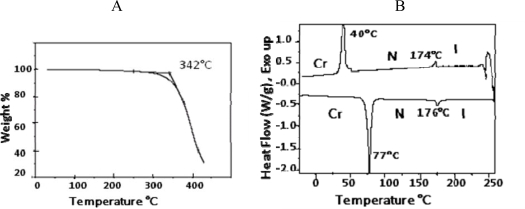
Thermogravemetric analysis and DSC graph of benzoic acid, 4-(4-pentenyloxy)-, 2-chloro-1,4-phenylene ester (**4**).

As shown in Scheme 2, mesogen **4** was synthesized by esterification of 2-chlorohydroquinone with 4-(4-pentenyloxy)benzoic acid (**2**), in the presence of DCC. ^1^H- and ^13^C-NMR spectra were consistent with the structure proposed for **4**. CH analysis results were within acceptable limits. TGA showed that the mesogen started to decompose at 342 °C (Figure 4 A). Figure 4 B shows DSC thermograms of compound **4.**

It displayed a nematic phase on both heating and cooling. The thermotropic behavior and the enthalpy values are given in Table 1. One can clearly see that higher energy is required for the transition from the crystalline to the nematic phase (7.13 kcal mol^−1^) while less energy is needed for the nematic to crystalline phase transition (0.47 kcal mol^−1^). This is due to the fact that larger energy is needed to disrupt both the positional and the orientational order of the crystalline phase; while less energy is needed to disrupt the orientational order of the nematic phase.

**Figure 5.**
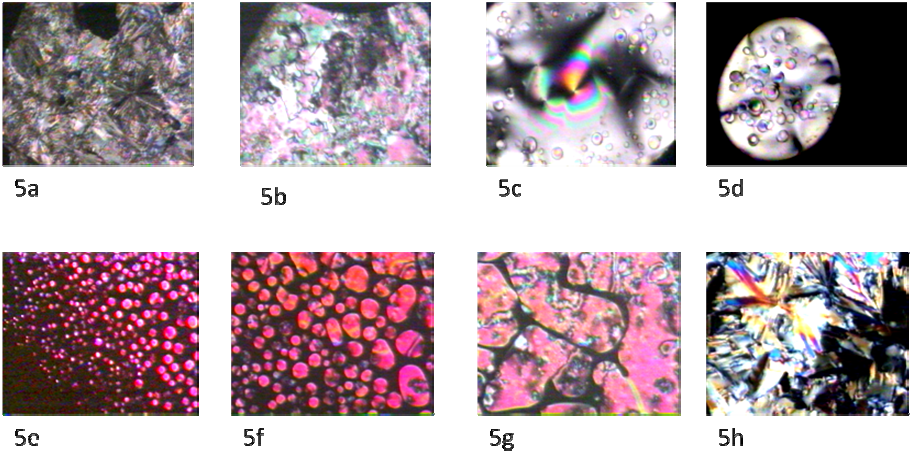
(5a) Crystalline texture of the mesogen; crossed polarizers, 25 °C, ×150. (5b) Nematic texture; crossed polarizers, 77.3 °C, ×150. (5c). Nematic–Isotropic transition; crossed polarizers.176 °C, ×150. (5d). Nematic –Isotropic Transition. The black domain is the isotropic phase with no optical activity; crossed polarizers,176 °C, ×150. (5e) Nematic droplets appear from the isotropic phase; crossed polarizers, 174 °C, ×150. (5f) Nematic droplets starts to coalesce to form the nematic texture; crossed polarizers, 174 °C, ×150. (5g) Nematic domain becomes more apparent and threadlike texture is appearing; crossed polarizers, 174 °C, ×150. (5h) Crystalline texture; crossed polarizers, below 40 °C, ×150.

Mesophase identification was achieved by optical polarized light microscopic observations. Figure 5 ailluastrates crystalline phases of mesogen **4**, with magnification of 150x under crossed polarizers. By bringing the temperature up to 77 °C, a crystalline to nematic transition was observed (Figure 5b). On further heating up to 174 °C, the nematic phase lost its birefringence and was transformed to an isotropic phase (Figures 5c and 5d). By cooling the isotopic phase at 10 °C/min, nematic droplets began to appear and coalesce (Figures 5e and 5f), forming a nematic texture (Figure 5g). The texture color change is due to the sensitivity of the birefringent nematic mesophase to temperature. Finally, when the temperature was reduced to 40 °C, a crystalline texture formed (Figure 5h).

#### 2.2.2. Benzoic Acid, 4-(4-Pentenyloxy)-, 2-Acetyl-1,4-phenylene Ester (**5**)

**Figure 6.**
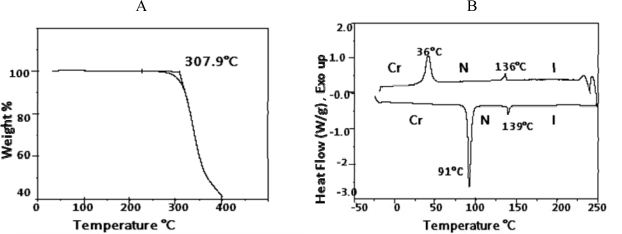
(6A) Thermogravemetric analysis of benzoic acid, 4-(4-pentenyloxy)-, 2-acetyl-1,4-phenylene ester (**5**). (6B) DSC thermograms for benzoic acid, 4-(4-pentenyloxy)-, 2-acetyl-1,4-phenylene ester (**5**).

Mesogen **5** was synthesized according to Scheme 2 by esterification of 2-hydroxyacetophenone with **2** in presence of DCC. A 1:10 mixture (by volume) of anhydrous acetone with methylene chloride was used in the reaction to insure complete miscibility of 2-hydroxyacetophenone. ^1^H- and ^13^C-NMR spectra were consistent with the structure suggested for **5**. CH analysis results were within acceptable limits. The thermogravimetric analysis showed that the mesogen began decomposing at 307 °C, as can be seen from Figure 6a. Figure 6B shows DSC thermograms of compound **5**. One can clearly see the presence of a nematic phase on both heating and cooling. The thermotropic behavior and the enthalpy values are presented in Table 2.

**Figure 7.**
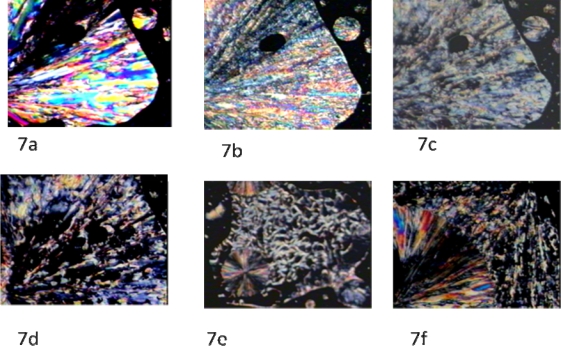
(7a) Initial crystalline texture of the mesogen; cross polarizers, 25 °C, ×150. (7b) Crystalline-Nematic transition; crossed polarizers, 90.5 °C, ×150. (7c) Nematic phase; 91 °C, ×150. (7d) Nematic-Isotropic transition upon heating. The black domain represents the isotropic phase; crossed polarizers, 139 °C, ×150. (7e) Nematic-Crystalline transition upon cooling; crossed polarizers 36 °C, ×150. (7f) Nematic-Crystalline transition; crossed polarizers 36 °C, ×150.

The mesophase identification was achieved by optical polarized light microscopic observations. Figures 7 shows crystalline phases for mesogen **5**. Upon heating, a phase transition occurred and a nematic texture was observed (Figures 7a and 7b). When the temperature was brought up to 139 °C, an isotropic phase was observed (Figure 7c). Upon cooling of the isotropic phase, a nematic texture was observed, followed by crystalline phase appearance at 36 °C (Figures 7d, 7e, and 7f).

#### 2.2.3. Benzoic Acid, 4-(4-pentenyloxy)-, 2-Methoxy-1,4-phenylene Ester (6): Synthesis

As shown in Scheme 2, mesogen **6** was synthesized by esterification of 2-methoxyhydroquinone with **2** in the presence of DCC. ^1^H- and ^13^C-NMR spectra were consistent with the structure for **6**, and CH analysis results were within acceptable limits.

**Figure 8.**
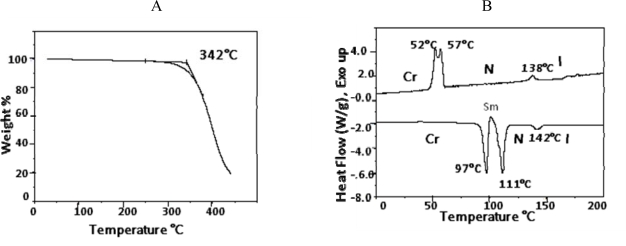
(8a) Thermogravemetric analysis of benzoic acid, 4-(4-pentenyloxy)-, 2- methoxy-1,4-phenylene ester (**6**). (8b) DSC thermograms for benzoic acid, 4-(4-pentenyloxy)-, 2- methoxy-1,4-phenylene ester (**6**).

TGA data indicated that compound **6** began to decompose at 342 °C (Figure 8a). Figure 8b represents DSC thermograms of compound **6**. Smectic and nematic phases were observed on both heating and cooling. The thermotropic behavior and the enthalpy values are listed in Table 3.

**Figure 9.**
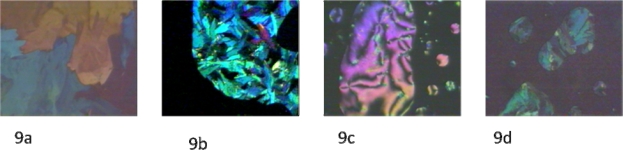
(9a) Crystalline-Smectic phase transition state; crossed polarizers, 97 °C, ×150. (9b) Smectic phase (smectic A) with broken fan-shape texture; crossed polarizers, 97 °C,×150. (9c) Nematic schlieren texture with point singularities;crossed polarizers, below 138 °C, ×150. (9d) Smectic phase upon cooling exhibited broken fans; crossed polarizers, 57°C,×150.

Mesophase identification of compound **6** was achieved by optical light polarizedmicroscopic observations. When the temperature was raised to 97 °C, a change was observed under the microscope (Figure 9a). Figure 9b shows a smectic phase (likely smectic A) with focal domains and broken fans texture, a characteristic features of smectic phases. A transition from the smectic to nematic phase was observed at 111 °C and a nematic to isotropic transition was observed at 142 °C. Upon cooling the isotropic phase, nematic droplets appeared from the isotropic phase; these droplets coalesce to form a nematic phase texture, which results in a point of defects and two and four brushes;see Figures 9c. On further cooling to 57 °C, the smectic phase has appeared for a very short period of time, often being undetected by DSC or under the microscope (see Figure 9d). Finally, at 36 °C, a crystalline phase was observed.

#### 2.2.4. Benzoic Acid, 4-(4-Pentenyloxy)-, 2-Methyl-1,4-phenylene Ester (**7**)

**Figure 10.**
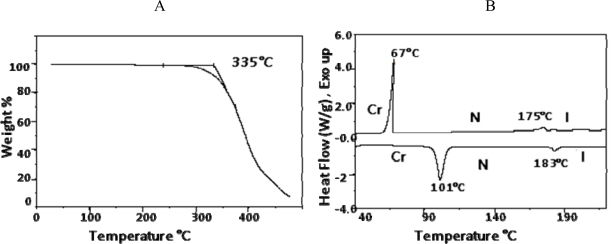
(10A) Thermogravemetric analysis of benzoic acid, 4-(4-pentenyloxy)-, 2-methyl-1,4-phenylene ester (**7**). (10B) DSC thermograms for benzoic acid, 4-(4-pentenyloxy)-, 2- methyl-1,4-phenylene ester (**7**).

As shown in Scheme 2, mesogen **7** was synthesized by esterfication of 2-methylhydroquinone with **2** in the presence of DCC. ^1^H- and ^13^C-NMR spectra were consistent with the structure for **7**. The ^1^H-NMR spectrum was identical to that reported in the literature [26]. TGAindicated that mesogen **7** started to decompose at 335 °C, as seen from Figure 10a. Figure 10b shows the DSC thermograms of compound **7**, revealing nematic phases on both heating and cooling. The thermotropic behavior and the enthalpy values are listed in Table 4.

**Figure 11.**
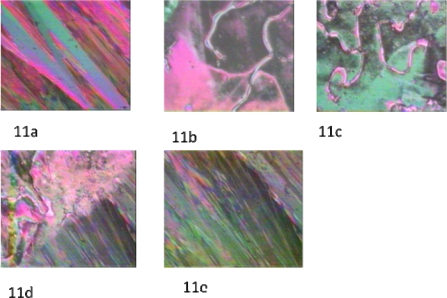
(11a) Crystalline texture of the mesogen; crossed polarizers, 25 °C, ×150. (11b) Nematic thread-like texture; crossed polarizers,101 °C, ×150. (11c) Nematic thread-like texture; crossed polarizers, below 175 °C, ×150. (11d) Nematic-Crystalline phase transition; crossed polarizers, 67 °C, ×150. (11e) Crystalline phase; crossed polarizers, below 67 °C, ×150.

Texture identification for mesogen **7** has been achieved by optical polarized light microscopic observations. Figure 11a presents the crystalline phase of the mesogen. Upon heating up to 101 °C, a phase transition was observed, exhibiting thread-like nematic texture (Figure 11b). On further heating to 131 °C, the nematic to isotropic phase transition occurred. Upon cooling, nematic droplets emerged and coalesced to form a nematic texture (Figure 11c). The nematic texture was characterized by its thread-like texture and line defects. On further cooling to 67 °C, the crystalline phase began replacing the nematic phase (Figures 11d and 11e).

### 2.3. The Effect of Lateral Substituent Size on the Clearing Point

**Figure 12.**
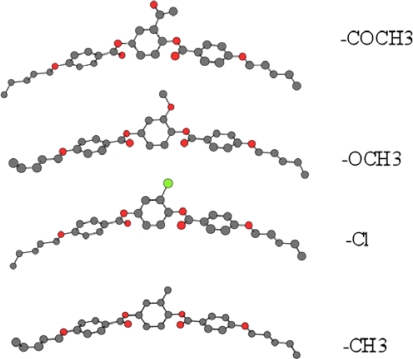
Lateral substitution in the mesogen.

It was found that the nematic-isotropic (T_N-I_) transition temperatures decreased by increasing the lateral substituent size (Figure 12) in the following order:

$$-{\text{COCH}}_{3}(139\;{}^{\circ}\text{C})\;<\;-{\text{OCH}}_{3}(142\;{}^{\circ}\text{C})\;<\;-\text{Cl}(176\;{}^{\circ}\text{C})\;<\;-{\text{CH}}_{3}(183\;{}^{\circ}\text{C})$$

Figure 12 shows the size of lateral substituents in the mesogens, as calculated by MM2 energy minimization computational analysis using the Cambridge Scientific Chem 3D program. As anticipated, the more bulky the substitutent, the lower the packing density of the molecules, and the weaker the Van der Waals intermolecular forces of attraction. This results in decreased clearing temperatures with increased size of the lateral substituents. This molecular design strategy provides an excellent method to modulate the clearing temperature of this class of liquid crystalline materials.

## 3. Experimental Section

### 3.1. Materials

5-Bromo-1-pentene (97%), 2,5-dihydroxyacetophenone (97%), 2-methoxyhydroquinone (97%), potassium carbonate (99+%) were used as received from Lancaster. 4-(Dimethylamino)pyridine (DMAP, 99%), 1,3-dicyclohexylcarbodiimide (DCC, 99%), methylhydroquinone (99%), ethyl hydroxybenzoate (99%) were used as received from Aldrich. Chlorohydroquinone (94+%) was used as received from Alfa Aesar. MN-Aluminum Oxide, neutral (manufactured by Machery Nagel & Co) was used as received. Commercial methylene chloride was distilled from lithium aluminum hydride and stored over molecular sieves in a sealed container. THF was dried by distillation over sodium, and stored over molecular sieves in a sealed container. HPLC grade acetonitrile was dried using molecular sieves and stored in a sealed container. All other reagents and solvents were commercially available, unless noted otherwise.

### 3.2. Instrumentation

Thermogravimetric analysis (TGA) was performed using a TGA 2050 thermogravimetric analyzer (*TA* Instruments) at a heating rate of 20 ° C/min. The thermotropic behavior of all compounds was determined by a combination of differential scanning calorimetry (DSC) and polarized optical microscopy. A *TA* Instruments 2920 differential scanning calorimeter (DSC) was used to determine the thermal transitions that were read as the maximum or minimum of the endothermic or exothermic peaks, respectively. All heating and cooling rates were 10 ° C/min. Unless stated otherwise, thermal transitions were read from reproducible second or later heating scans and first or later cooling scans, respectively. An Olympus BH-2 polarized optical microscope (magnification ×150), equipped with a Mettler FP52 hot stage, was used to detect and image phase transitions. Images were captured using a video camera and frame grabber software. Thin samples were prepared by melting a minimum amount of compound between a clean glass slide and a cover slip. ^1^H- and ^13^C-NMR analyses were performed in CDCl_3_ (referenced to TMS at δ 0.0 ppm) on a Varian Gemini spectrometer (300 MHz for ^1^H and 75 MHz for ^13^C).

### 3.3. Synthesis

#### 3.3.1. Preparation of the K_2_CO_3_ · Al_2_O_3_ Catalyst

Potassium carbonate (103 g, 0.74 mmol) was mixed with neutral aluminum oxide (150 g, 1.47 mmol) in distilled water (300 mL). The mixture was stirred and heated to 50 ° C for 1 h. The water was evaporated, and the resulting catalyst was activated overnight in the oven at 120 ° C.

#### 3.3.2. Ethyl 4-(4-Pentenyloxy)benzoate [27]

A solution of 5-bromo-1-pentene (12.6 g, 85 mmol) was added dropwise to a refluxing solution of ethyl 4-hydroxybenzoate (15 g, 90 mmoL) and alumina-supported potassium carbonate (the catalyst). After 5 h of reflux, the reaction mixture was poured into water and extracted three times with methylene chloride (400 mL total). The organic layer was filtered to remove any traces of the catalyst. The solvent was removed on a rotary evaporator *in vacuo* to yield 19.2 g (97%) of ethyl 4-(4-pentenyloxy) benzoate as a slightly yellowish oil. ^1^H-NMR (δ ppm TMS, CDCl_3_): 1.38 (t, CO_2_CH_2_C*H*_3_), 1.90 (m, C*H*_2_CH_2_O), 2.25 (q, C*H*_2_CH=), 4.02 (t, OC*H*_2_), 4.34 (q, CO_2_C*H*_2_), 5.05 (m, =C*H*_2_), 5.85 (m, =C*H*), 6.90 (d, 2 aromatic H ortho to OR), 7.98 (d, 2 aromatic H ortho to CO_2_R’); ^13^C-NMR (δ ppm TMS, CDCl_3_): δ 14.7, 28.6, 30.3, 60.8, 67.5, 114.1,115.5, 122.9, 131.6, 137.7, 162.9, 166.7.

##### 3.3.3. 4-(4-Pentenyloxy)benzoic Acid (**1**) [27]

Ethyl 4-(4-pentenyloxy)benzoate (19.2 g, 82 mmol) and potassium hydroxide (15 g, 0.27 mol), dissolved in ethanol (50 mL) and water (100 mL), were refluxed for 6 h. After being cooled to room temperature, the solution was acidified to pH 2 with concentrated HCl. The resulting precipitate was collected and recrystallized from ethanol, affording 15.2 g (90%) of 4-(4-pentenyloxy)benzoic acid (**1**) as white shiny crystals. ^1^H-NMR (δ ppm TMS, CDCl_3_): 1.92 (m, C*H*_2_CH_2_O), 2.24 (q, C*H*_2_CH=), 4.04 (t, OC*H*_2_), 5.05 (m, =C*H*_2_), 5.86 (m, C*H*=), 6.93 (d, 2 aromatic H ortho to OR), 8.05 (d, 2 aromatic H ortho to CO_2_H); ^13^ C-NMR (δ ppm TMS, CDCl_3_): δ 28.2, 30.0, 67.3, 114.0, 115.2, 121.3, 132.1, 137.3, 163.3, 171.8.

#### 3.3.4. Benzoic Acid, 4-(4-Pentenyloxy)-, 2-Chloro-1,4-phenylene Ester (**4**)

4-(4-Pentenyloxy)benzoic acid (**1**) (1.04 g, 5.1 mmol), chlorohydroquinone (0.4 g, 2.8 mmol), DMAP (0.05 g, 0.4 mmol), and DCC (1.04 g, 5.1 mmol) in dry CH_2_Cl_2_ (15 mL) were stirred at room temperature for 48 h. The reaction mixture was then extracted with water and methylene chloride. The precipitate of the dicyclohexylurea by-product was filtered off and discarded. The solvent was removed using a rotary evaporator. The resulting solid was recrystallized from ethanol, yielding 1.2 g (70%) of benzoic acid, 4-(4-pentenyloxy)-, 2-chloro-1,4-phenylene ester (**4**) as white crystals. ^1^H-NMR (δ ppm TMS, CDCl_3_): 1.95 (m, C*H*_2_CH_2_O, 4 H), 2.26 (m, C*H*_2_CH=, 4 H), 4.06 (t, OC*H*_2_, 4 H), 5.05 (m, =C*H*_2_, 4 H), 5.85 (m, =C*H*, 2 H), 6.97(dd, 4 aromatic H ortho to OR), 7.17 (m, 3 aromatic H of central ring), 8.13 (dd, 4 aromatic H ortho to CO_2_Ar);^13^ C-NMR ((δ ppm TMS, CDCl_3_): δ 28.6, 30.4, 67.778, 114.6, 115.7, 121.4, 124.0, 124.4, 127.2, 132.7, 137.7, 144.3, 148.2, 163.7, 164.1; Anal. Calcd. for C_30_H_29_ClO_6_: C, 69.16; H, 5.61; Cl, 6.80, found: C, 69.00; H, 5.66; Cl, 6.80.

#### 3.3.5. Benzoic Acid, 4-(4-Pentenyloxy)-, 2-Acetyl-1,4-phenylene Ester (**5**)

4-(4-Pentenyloxy)benzoic acid (**1**, 1.04 g, 5.1 mmol), 2,5 dihydroxyacetophenone (0.4 g, 2.6 mmol), DMAP (0.05 g, 0.4 mmol), DCC (1.04 g, 5.1 mmol), dry CH_2_Cl_2_ (15 mL), and dry acetone (2 mL), were stirred at room temperature for 48 h. Extraction of the product was carried out with water and methylene chloride. The precipitate of dicyclohexylurea by-product was filtered off and discarded. The solvent was removed using a rotary evaporator. The resulting solid was recrystallized from ethanol, providing 0.69 g (50%) of benzoic acid, 4-(4-pentenyloxy)-, 2-acetyl-1,4-phenylene ester (**5**) as white crystals. ^1^H-NMR (δ ppm TMS, CDCl_3_): 1.94 (m, C*H*_2_CH_2_O, 4 H), 2.27 (m, C*H*_2_CH=, 4 H), 2.54 (s, *O= CCH3*), 4.06 (t, OC*H*_2_, 4 H), 5.05 (m, = C*H*_2_, 4 H), 5.85 (m, = C*H*, 2 H), 6.97 (dd, 4 aromatic H ortho to OR), 7.46 (m, 3 aromatic H of central ring), 8.13 (dd, 4 aromatic H ortho to CO_2_Ar);^13^C-NMR (δ ppm TMS, CDCl_3_): δ 23.1, 28.3, 30.2, 68.1, 114.3, 115.8, 120.2, 123.5, 125.5, 127.1, 132.3, 139.6, 147.3, 148.6, 159.9, 160.5, 196.8; Anal. Calcd. for C_32_H_32_O_7_: C, 72.70; H, 6.10; found: C, 72.69; H, 6.16.

#### 3.3.6. Benzoic Acid, 4-(4-Pentenyloxy)-, 2-Methoxy-1,4-phenylene Ester (**6**)

4-(4-Pentenyloxy)benzoic acid (**1**, 1.04 g, 5.1 mmol), 2-methoxyhydroquinone (0.36 g, 2.56 mmol), DMAP (0.05 g, 0.4 mmol), and DCC (1.04 g, 5.1 mmol) in dry CH_2_Cl_2_ (15 mL) were stirred at room temperature for 48 h. The product was extracted with water and methylene chloride. The precipitate of dicyclohexylurea by-product was filtered off and discarded. The solvent was removed using a rotary evaporator. The resulting solid was recrystallized from ethanol, resulting in 0.66 g (50%) of benzoic acid, 4-(4-pentenyloxy)-, 2-methoxy-1,4-phenylene ester (**6**) as white crystals. ^1^H-NMR (δ ppm TMS, CDCl_3_): 1.95 (m, C*H*_2_CH_2_O, 4 H), 2.24 (m, C*H*_2_CH=, 4 H), 3.80 (s, *OCH_3_*) 4.06 (t, OC*H*_2_, 4 H), 5.05 (m, =C*H*_2_, 4 H), 5.86 (m, =C*H*, 2 H), 6.89 (dd, 4 aromatic H ortho to OR), 7.19 (m, 3 aromatic H of central ring), 8.13 (dd, 4 aromatic H ortho to CO_2_Ar);^13^C-NMR (δ ppm TMS, CDCl_3_): δ 28.2, 30.0, 56.0, 67.3, 106.6, 113.4, 114.1, 115.3, 121.2, 123.0, 132.2, 137.3, 149.0, 151.6, 163.2, 164.3. Anal. Calcd. for C_31_H_32_O_7_: C, 72.03; H, 6.24; found: C, 72.20; H, 6.22.

#### 3.3.7. Benzoic Acid, 4-(4-Pentenyloxy)-, 2-(Methyl)-1,4-phenylene Ester (**7**) [26]

4-(4-Pentenyloxy)benzoic acid (**1**, 1.12 g, 5.4 mmol), methylhydroquinone (0.7 g, 5.6 mmol), DMAP (0.07 g, 0.55 mmol), and DCC (1.5 g, 7.3 mmol) in dry CH_2_Cl_2_ (15 mL) were stirred at room temperature for 48 h. The product was extracted with mixture of water and methylene chloride. The precipitate of dicyclohexylurea by-product was filtered off and discarded. The solvent was removed using a rotary evaporator. The resulting solid was recrystallized from mixture of ethanol (80%) and toluene (20%) to yield 1.8 g (65%) of benzoic acid, 4-(4-pentenyloxy)-, 2-(methyl)-1,4-phenylene ester (**7**) as white crystals. ^1^H-NMR (δ ppm TMS, CDCl_3_): 1.86 (m, C*H*_2_CH_2_O, 4 H), 2.17 (s, *-CH_3_*) 2.21 (m, C*H*_2_CH=, 4 H), 3.98 (t, OC*H*_2_, 4 H), 4.98 (m, =C*H*_2_, 4 H), 5.78 (m, =C*H*, 2 H), 6.89 (dd, 4 aromatic H ortho to OR), 7.03 (m, 3 aromatic H of central ring), 8.06 (dd, 4 aromatic H ortho to CO_2_Ar); ^13^C-NMR (δ ppm TMS, CDCl_3_): δ 16.9, 28.6, 30.4, 67.7, 114.5, 115.7, 120.3, 121.7, 123.1, 124.3, 132.3, 137.7, 147.2, 148.6, 163.6, 164.9.

## 4. Conclusions

A series of novel calamitic liquid crystalline compounds that exhibit a broad nematic liquid crystalline range was synthesized and characterized. These mesogens contain reactive double bonds on the termini of the aliphatic chains, functionality that may serve to incorporate these liquid crystalline moieties into a polymeric materials in future research (*e.g.*, formation of nematic elastomers). DSC investigations revealed the presence of liquid crystal phases by detecting the enthalpy change associated with the phase transition; the amount of energy released or absorbed can be used as a preliminary detection and identification of the liquid crystalline phase. Therefore, lower energy transitions are typically associated with the nematic phase; while, higher energy transitions are normally associated with smectic and crystalline phases. The identification of the mesophases was accomplished with variable temperature optical polarized light microscopy. Phase texture (morphology) observations and transitions werefound to be consistent with the DSC results. Finally, the clearing points of the mesogens were greatly affected by the size of the lateral substituents, and decreased with increasing lateral substituent size, making this class of liquid crystals particular promising as their thermotropic properties can be readily modulated by changes in the molecular structure.

## Figures and Tables

**Table 1.** Phase transition temperatures and enthalpy changes of compound 4.

*Heating*		
**Phase Transition**	**Temperature °C**	**Δ H kJmol^−1^**

Cr-N	77.3	29.8
N-I	176	1.95

*Cooling*		

**Phase Transition**	**Temperature** °**C**	**Δ H kJmol^−1^**

I-N	174	1.95
N-Cr	40.0	25.2

I: isotropic, N:nematic, Cr: crystalline

**Table 2.** Phase transition temperatures and enthalpy changes of compound 5.

*Heating*		
**Phase Transition**	**Temperature °C**	**Δ H kJmol^−1^**

Cr-N	91.0	35.4
N-I	139	2.30

*Cooling.*		

**Phase Transition**	**Temperature** °**C**	**Δ H kJmol^−1^**

I-N	136	−2.36
N-Cr	36.0	−23.0

I: isotropic, N:nematic, Cr: crystalline.

**Table 3.** Phase transition temperatures and enthalpy changes compound **6**.

*Heating*		

**Phase Transition**	**Temperature °C**	**Δ H kJmol^−1^**

Cr-Sm	97.0	5.50
Sm-N	111	7.45
N-I	142	0.65

*Cooling*		

**Phase Transition**	**Temperature °C**	**Δ H kJmol^−1^**

I-N	138	−0.80
N-Sm	57	Coincided peaks
		Total= −8.1
Sm-Cr	52	Coincided peaks

I: isotropic, N:nematic, Sm: smectic, Cr: crystalline.

**Table 4.** Phase transition temperatures and enthalpy changes for compound **7**.

*Heating*		
**Phase Transition**	**Temperature °C**	**Δ H kJmol^−1^**

Cr-N	101	35.0
N-I	183	2.5

*Cooling*		

**Phase Transition**	**Temperature °C**	**Δ H kJmol^−1^**

I-N	175	−2.28
N-Cr	67	−31.1

I: isotropic, N:nematic, Cr: crystalline.
